# Evaluation of Dysfunctional HDL by Myeloperoxidase/Paraoxonase Ratio in Unexplained Infertility Patients Undergoing IVF/ICSI

**DOI:** 10.3390/jcm12041506

**Published:** 2023-02-14

**Authors:** Kadriye Erdoğan, Nazli Tunca Sanlier, Emine Utlu Özen, Süleyman Erol, Inci Kahyaoğlu, Salim Neselioglu, Özcan Erel, Serra Akar, Yaprak Engin Üstün

**Affiliations:** 1 Department of Obstetrics and Gynecology, Etlik Zübeyde Hanım Women’s Health Training and Research Hospital, Health Sciences University, 06830 Ankara, Turkey; 2 Department of Obstetrics and Gynecology, Ankara City Hospital, 06800 Ankara, Turkey

**Keywords:** unexplained infertility, dysfunctional high-density lipoprotein, myeloperoxidase, paraoxonase

## Abstract

The relationship between oxidative stress and unexplained infertility (UEI) has not been studied in detail. This is the first study to evaluate dysfunctional high-density lipoprotein (HDL) by the myeloperoxidase (MPO) and paraoxonase (PON) ratio to investigate the role of oxidative stress in UEI. Materials and Methods: Patients with UEI (study group, *n* = 40) and male factor infertility (control group, *n* = 36) were included in this prospective study. Demographics and laboratory assessments were analyzed. Results: Total dosages of gonadotropin were higher in UEI when compared to the control group (*p* = 0.033). Number of Grade 1 embryos and the quality of blastocysts were lower in UEI than in the control group (*p* = 0.024, *p* = 0.020, respectively), whereas serum MPO/PON ratio was higher in UEI (*p* = 0.042). Stepwise linear regression analysis revealed that serum MPO/PON ratio levels could significantly predict the duration of infertility (*p* = 0.012). Conclusions: Serum MPO/PON ratio increased in patients with UEI, whereas the number of Grade 1 embryos and the quality of blastocysts decreased. Similar clinical pregnacy rates were found in both groups but the ET on day five is associated with higher clinical pregnancy rate in the male factor infertility.

## 1. Introduction

UEI is the failure to conceive following one year of unprotected intercourse without an identifiable reason [1]. If we simplify the diagnostic criteria of UEI, it can be listed as having normal ovulatory function and semen analysis, and at the minimum unilateral patency of the fallopian tube [2]. The diagnosis of UEI is usually made by excluding other existing causes. Investigating the cause of something unknown has made its research more intriguing, especially if it concerns more than 30% of infertile couples [3]. In order to avoid overdiagnosis and overtreatment, investigations on the causes of UEI have increased with a particular focus on the role of oxidative stress [4,5]. HDL consists of lipids (cholesterol, triglycerides, phospholipids) and proteins (apolipoproteins and enzymes). Apolipoprotein A (apoA)-I is a major protein component of HDL and constitutes approximately 70% of HDL. ApoA-II, an important HDL apolipoprotein, can interfere with apoA-I interaction with HDL. Other apoproteins can be transferred by HDL and these include apoE, apoCs, apoA-IV, apoA-V, apoJ, apoF, apoM, apoL1 [6]. Among the functions of HDL are its activation of nitric oxide and its anti-inflammatory, antioxidant and anti-apoptotic roles [7]. Under oxidative stress, HDL loses its anti-inflammatory properties and becomes pro-inflammatory (dysfunctional). Paraoxonase (PON) is one of the major HDL-associated proteins that acts as an anti-oxidative enzyme [8]. PON protects lipoproteins against oxidative stress by inhibiting LDL glycation and lipoprotein peroxidation. Also, PON passes from HDL to the cell membrane, then attaches to its external surface and makes it less susceptible to oxidative stress [9]. Myeloperoxidase (MPO) is a heme peroxidase and is produced from granular leukocytes [10]. When MPO binds to a specific region of helix 8 of apoA-I, it impairs its function. Disruption of apoA-I by MPO results in dysfunctional HDL and oxidation ensues [11]. MPO and PON are the major contributors of HDL function [12]. It was shown that HDL obtained from patient samples with elevated MPO/PON ratios demonstrated diminished anti-inflammatory activity. MPO/PON ratio impacts the activity of HDL and a significant direct association has been found between the MPO/PON ratio and HDL activity [13].

Considering that the functions of lipoproteins are impaired by MPO and improved by PON, examining the activities of these two enzymes can enable a better understanding of oxidative stress. Thus, this study is the first to evaluate dysfunctional HDL though the MPO and PON ratio to elucidate the impact of oxidant-antioxidant mechanisms in the etiology of UEI. As such, the effect of oxidative stress was further investigated in the management and outcomes of patients undergoing in vitro fertilization (IVF)/intracytoplasmic sperm injection (ICSI).

## 2. Materials and Methods

### 2.1. Study Design

**Figure 1:**
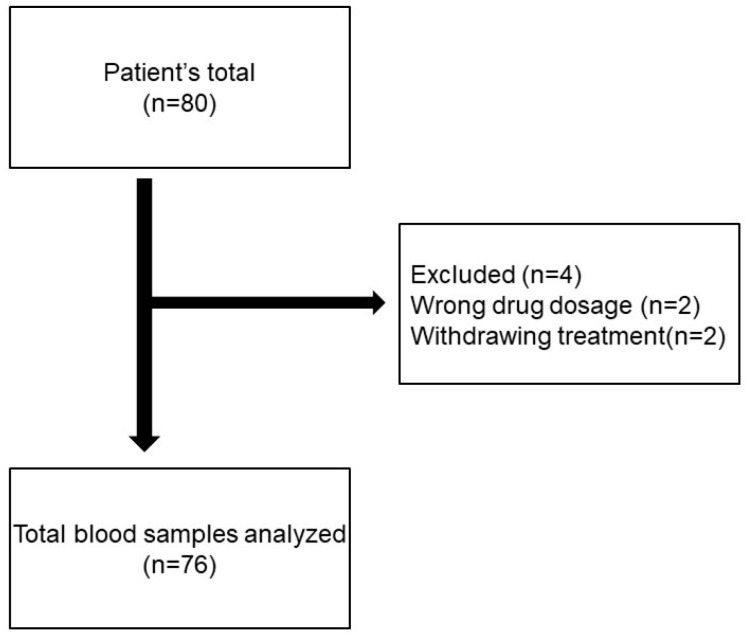
The study flow chart.

A total of 80 patients were initially included in the study between May 2022 and November 2022. Two patients were excluded from the study because of their use of medications different from the ones that were prescribed. Two other patients were excluded from the study because the couples refused ongoing treatment. Finally, this prospective study was carried out with a total of 76 women, who were treated at the assisted reproduction clinics of Etlik Zübeyde Hanım Women’s Health Training and Research Hospital of Ankara, Turkey. The study flow chart is given in Figure 1. The research protocol was approved by the Local Ethical Committee (20.04.2022 2022/59). Patients who had a normal ovulatory function, semen analysis, and at the minimum a single patent fallopian tube were considered as having UEI and formed the study group (*n* = 40). The control group consisted of patients who were diagnosed with mild-moderate male factor infertility (*n* = 36).

Exclusion criteria included: a body mass index (BMI) > 35 kg/m^2^, age < 18 and > 40 years, a history of smoking, acute infection (within 14 days), chronic inflammatory autoimmune disease, any systemic or endocrine disease, multiple embryo transfers, frozen-thaw cycles, moderate or severe ovarian hyperstimulation syndrome (OHSS), use of any medication including vitamin supplements and couples with severe male infertility including azoospermia and severe oligoasthenospermia.

Demographic characteristics (maternal and paternal ages, BMI), number of cycles, total dosages of gonadotropin, duration of infertility and stimulation were recorded. Basal serum hormone levels on day three (D3) of the cycle, antral follicle count, number of retrieved and metaphase II (MII) oocytes, Grade 1-2-3 embryos [14], blastocyst quality scoring (BQS) [15], day of embryo transfer (ET), clinical pregnancy after fresh transfer cycles and serum MPO/PON ratio were recorded.

### 2.2. Controlled Ovarian Stimulation (COS)

Patients enrolled in our study received a gonadotropin-releasing hormone (GnRH) antagonist protocol for COS [16], and human Chorionic Gonadotrophin (hCG) trigger (Ovitrelle^®^, Merck, Germany) was used when at least three follicles were measured over 17–18 mm in diameter. Oocyte pick up (OPU) was performed 34–36 h later with the guidance of transvaginal ultrasonography. Oocytes were incubated as cumulus complex at 37 °C 5% CO_2_ and 5% O_2_ for two hours before denudation for ICSI. Denudation was completed by both hyaluronidase (Hyase 10X, Vitrolife, Sweden) and the mechanical technique. Fertilization was confirmed by the presence of two pronuclei 18–20 h after the ICSI. One step culture protocol (G-TL, Vitrolife, Sweden) used for the embryo culture under oil at 37 °C 5% CO_2_ and 5% O_2_ (Miri, ESCO Medical, Turkey) was performed [14]. Embryos were graded according to the morphological criteria of Gardner and Schoolcraft [17] and BQS was performed based on the criteria established by Rehman et al. [15]. The embryos were transferred to the uterus on day three or day five. Luteal phase support was provided with intramuscular (100 mg daily) progesterone (Progestan^®^, Koçak Pharma, Turkey) and oral (10 mg three times a day) dydrogesterone (Duphaston^®^, Abbott, Turkey) until 12 weeks gestational age in all patients. Observation of the gestational sac with transvaginal ultrasonography was accepted as clinical pregnancy.

### 2.3. Collection of Blood Samples

On the OPU day, 5 mL of blood was withdrawn from the antecubital vein after overnight fasting and samples were centrifuged at 1500 rpm for 10 min. The sera were separated and stored at −80 degrees until serum MPO/PON ratio was measured.

### 2.4. Measurement of PON Activity

The paraoxon hydrolysis rate was calculated from the rise of spectroscopic absorbance at 412 nm. This was used to calculate PON activity. In order to determine the total amount of p-nitrophenol production, molar absorptivity coefficient for a pH of 8.5, which was 18,290 M^−1^ cm^−1^ was employed. PON activity was expressed in U/L serum [18].

### 2.5. Measurement of MPO Level

Measurement of myeloperoxidase levels was performed using commercially available Human Myeloperoxidase ELISA kits (Cayman Chemical, Ann Arbor, MI, USA).

For the Human Myeloperoxidase ELISA kit (Item no:501410), the detection range in the analysis of the samples was stated as 0.2–10 ng/mL and the sensitivity as 0.2 ng/mL.

Within-run and between-run coefficient of variation (CV) values for the kit were 3.83% and 8.13%, respectively.

### 2.6. Determination of Dysfunctional HDL

The MPO/PON ratio was found to significantly impact and alter the activity of HDL. This ratio was also shown to correlate directly with HDL function. Therefore, MPO and PON enzyme levels should be measured to determine serum dysfunctional HDL levels [13].

### 2.7. Statistical Analysis

After determining whether the data were normally distributed using histograms, plots and the Shapiro–Wilk test, descriptive analyses were given as mean and standard deviation (mean ± SD) and the median (minimum-maximum) for normally and non-normally distributed variables, respectively. Independent samples t-test and Mann–Whitney U test were used to compare parametric and non-parametric group variables, respectively. Pearson’s correlation was used to investigate the potential association between group variables. Independent predictors of the duration of infertility were determined using stepwise linear regression analysis. *p*-value < 0.05 was accepted as statistically significant. All analyses were conducted by SPSS 20.0 (IBM Corp., Armonk, NY, USA).

## 3. Results

Totally 76 women were included in the study. Baseline features of participants were given in Table 1. We found no significant differences in maternal and paternal age and BMI in the UEI group than in the control group (*p* > 0.05) (Table 1). The D3 follicle-stimulating hormone (FSH), luteinizing hormone (LH) and estradiol (E2) concentrations statistically were similar between the two groups (*p* > 0.05) (Table 1). There was no significant difference in total antral follicle count, number of cycles, duration of infertility and stimulation between the groups (*p* > 0.05) (Table 1). Total dosages of gonadotropin were higher in UEI when compared to the control group (*p* = 0.033) (Table 1).

There was no significant difference in retrieved and MII oocyte counts in the UEI group than in the control group (*p* > 0.05) (Table 2). The number of Grade 2 and 3 embryos statistically were similar between the two groups (*p* > 0.05) (Table 2). We found no significant difference in clinical pregnancy rates between the groups (*p* > 0.05) (Table 2).

Number of Grade 1 embryos and BQS were lower in UEI than in the control group (*p* = 0.024) (*p* = 0.020), whereas serum MPO/PON ratio, MPO and PON were higher in UEI as compared to the control group (*p* = 0.042) (Table 2). The serum MPO/PON ratio was significantly higher in the UEI group (1.04 ± 0.65) than in the control group (0.63 ± 0.32) (*p* = 0.042). The MPO level was significantly higher in the study group (125.18 ± 79.48) than in the control group (76.44 ± 25.24) (*p* = 0.010). The PON level was higher in the UEI group (117.55) than in the control group (102.4) (*p* = 0.015) (Table 2).

In the control group, following day three ET, there was no clinical pregnancy and following day five ET, clinical pregnancy rate was 66.7% (Table 3). In the study group, the clinical pregnancy rates were not significantly different according to the day of ET (Table 3).

**Figure 2:**
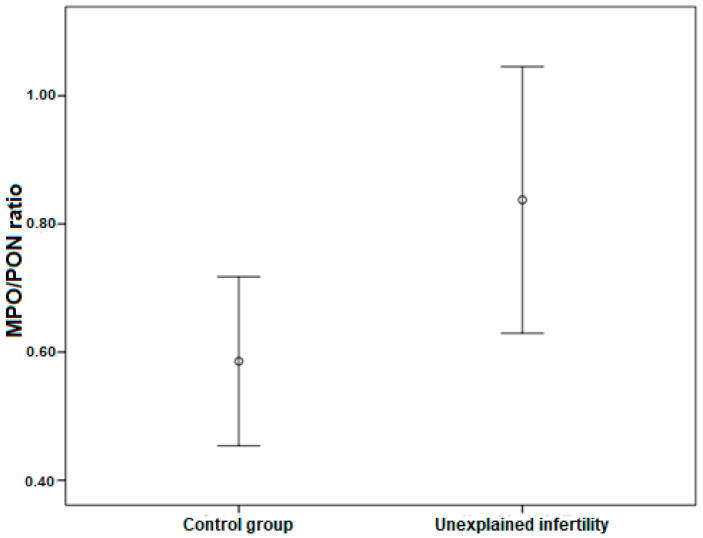
The alteration of serum MPO/PON ratio in control and unexplained infertility groups ($\bar{x}\;\pm \;\sigma \bar{x}$).

The alterations of serum MPO/PON ratio in the control and UEI groups ($\bar{x}$ ± σ$\bar{x}$) are presented in Figure 2.

A significant direct correlation was observed between serum MPO/PON ratio and the duration of infertility (r = 0.389; *p* = 0.041). Stepwise linear regression analysis is given in Table 4. It revealed that serum MPO/PON ratio could significantly predict the duration of infertility (*p* = 0.012) (Table 4).

## 4. Discussion

To the best of our knowledge, the present study was the first to evaluate serum MPO/PON ratio for determining the role of oxidative stress in UEI patients undergoing IVF/ICSI. In this study, we found that serum MPO/PON ratio increased in UEI, while the number of Grade 1 embryos and the quality of blastocysts decreased in UEI.

Reactive oxygen species (ROS) are products of cell metabolism that carry out physiological roles such as the regulation of the activity of transcription factors, membrane receptors, structural proteins and ion channels. However, when ROS levels are elevated, the antioxidant mechanisms become inadequate in neutralizing oxidative stress [19]. The development of oocytes in the ovary, ovulation, the persistence and the regression of corpus luteum were regulated by the oxidant-antioxidant equilibrium. Normally, HDL has antioxidant, anti-inflammatory and anti-atherogenic effects, and it provides critical lipid nutrients to the oocytes for maintenance of cellular cholesterol balance, which is essential for the normal reproductive pathway. This pathway is disrupted by the alteration of HDL into the dysfunctional form under oxidative stress. Oxidative stress has been associated with UEI in previous studies [20,21,22]. Şentürk et al. reported that serum total oxidant status could have a partial role in the etiopathogenesis of UEI and oxidative stress index was increased in UEI patients [23]. Also, Verit et al. demonstrated that UEI patients had an atherogenic lipid profile with decreased HDL and with an increased risk for cardiovascular disease [24]. In the present study, serum MPO/PON ratio was higher in UEI than in the control group. In line with the results of this study, Pekel et al. found that the serum antioxidant capacity was impaired in UEI patients [25]. Contrarily, Younis et al. reported that the serum PON concentration did not change in UEI [26].

Oocyte quality is a major determinant in the achievement of pregnancies during an IVF treatment. Unfortunately, there are no decisive diagnostic tests for poor oocyte quality, in which case infertility may be classified as unexplained. The fact that young patients with normal ovarian reserve tests may surprisingly demonstrate poor oocyte quality is in agreement with the findings of the current study [27]. Although, ROS are produced within the follicle to excite ovulation and oocyte maturation; the excessive production of ROS may give rise to an increased risk of oocyte pathology. Consequently, elevated levels of ROS impair the developmental competence of oocytes and it is not counterintuitive that the use of exogenous antioxidants became more popular to avoid the negative impact of elevated ROS [28]. In the present study, number of Grade 1 embryos and BQS were lower in UEI. Based on a literature review, Bedaiwy et al. revealed that impaired embryo development was found in UEI patients whereas, Sentürk et al. did not find any association between serum oxidative markers and embryo quality [23,29].

It is not clear how ROS influence FSH signaling in recruited follicles. In the current study, total dosages of gonadotropin were higher in the UEI group, where oxidative stress was high. In contrast, Velthut et al. declared that more oocytes were collected by using less FSH per retrieved oocyte in patients with higher oxidative stress markers [30]. We found similar clinical pregnancy rates and different oxidative stress levels in the groups. This is probably due to the limited number of participants in each group. Furthermore, in the present study, the clinical pregnancy rate was higher in the control group following day five ET. A study by Hatırnaz et al. demonstrated that ET on days three and five resulted in similar pregnancy outcomes while in another study Zech et al. showed that the day five transfer of embryos resulted in better pregnancy outcomes compared to day three ET [31,32]. It is unknown whether the association between serum MPO/PON ratio and the duration of infertility is causal or not. This uncertainty can be solved with long term studies.

## 5. Conclusions

Serum MPO/PON ratio increased in patients with UEI, whereas the number of Grade 1 embryos and the quality of blastocysts decreased in UEI. Similar clinical pregnancy rates were found in both groups but the ET on day five is associated with higher clinical pregnancy rate in the male factor infertility. Larger studies will provide more reliable results.

## Figures and Tables

**Table 1:** Baseline features of participants.

Parameters	Control Group (*n* = 36)	Study Group (*n* = 40)	*p* Values
Maternal age (years) *	31.92 ± 3.99	32.2 ± 3.85	0.494
Paternal age (years) *	33.06 ± 5.81	34.78 ± 4.19	0.196
BMI (kg/m^2^)	25.48 ± 5.05	25.83 ± 4.16	0.203
D3 FSH (mIU/mL) **	6.62 (5.63–13.2)	6.18 (4.7–8.6)	0.925
D3 LH (mIU/mL) *	6.87 ± 3.55	7.03 ± 3.08	0.181
D3 E2 (pg/mL) **	34.35 (12.9–70.2)	36.8 (20.8–263)	0.171
Total AF count **	13.5 (8–30)	11 (5–30)	0.06
Number of cycles **	2 (1–4)	2.5 (1–4)	0.163
Duration of infertility (month) *	64.5 ± 39.4	75 ± 22.14	0.058
Duration of stimulation (day) *	10 ± 1.21	10.3 ± 1.06	0.529
Total dosages of gonadotropin *	1792.71 ± 654.58	1863.75 ± 556.23	0.033 ***

* Values are mean ± standard deviation, ** Values are median(min–max), *** p-value of less than 0.05 was considered to be statistically significant. Abbreviations: BMI, body mass index; D3, day three; FSH, follicle-stimulating hormone; LH, luteinizing hormone; E2, estradiol; AF, antral follicle.

**Table 2:** Comparison of in vitro fertilization outcomes and laboratory data.

Parameters	Control Group (*n* = 36)	Study Group (*n* = 40)	*p* Values
Retrieved oocyte count **	8.5 (1–17)	7 (5–24)	0.337
MII oocyte count *	7.25 ± 4.48	6.4 ± 2.32	0.437
Number of Grade 1 embryo **	1 (0–6)	0.5 (0–2)	0.024 ***
Number of Grade 2 embryo **	0.5 (0–2)	1 (0–2)	0.752
Number of Grade 3 embryo **	0.5 (0–3)	0.5 (0–2)	0.888
Serum MPO/PON ratio *	0.63 ± 0.32	1.04 ± 0.65	0.042 ***
MPO *	76.44 ± 25.24	125.18 ± 79.48	0.010 ***
PON **	102.4 (82.27–880.76)	117.55 (95.4–159.12)	0.015 ***
BQS *	32.11 ± 11.82	18.63 ± 9.57	0.020 ***
Clinical pregnancy (%)	42.9%	50%	0.491

* Values are mean ± standard deviation, ** Values are median(min–max), *** p-value of less than 0.05 was considered to be statistically significant. Abbreviations: MII, metaphase II; MPO, myeloperoxidase; PON, paraoxonase; BQS, blastocyst quality scoring.

**Table 3:** Comparison of clinical pregnancy rate according to the day of embro transfer.

Parameters		Control Group (*n* = 36)	Study Group (*n* = 40)	*p* Values
Clinical pregnancy (%)	Day 3 ET	0	50	0.725
	Day 5 ET	66.7	50	0.028 *

* p-value of less than 0.05 was considered to be statistically significant. Abbreviations: ET, embryo transfer.

**Table 4:** Stepwise linear regression analysis for the duration of infertility.

	Model I (R^2^ = 0.068)		Model II (R^2^ = 0.145)		Model III (R^2^ = 0.262)	
	β	*p*	β	*p*	β	*p*
Serum MPO/PON ratio	0.291	0.030 *	0.297	0.025 *	0.314	0.012 *
BMI			0.259	0.049 *	0.356	0.006 *
Basal D3 FSH					0.356	0.006 *

* *p*-value of less than 0.05 was considered to be statistically significant. Abbreviations: MPO, myeloperoxidase; PON, paraoxonase; BMI, body mass index; D3, day 3; FSH, follicle-stimulating hormone.

## Data Availability

Data can be made accessible upon demand.

## Abbreviations

Unexplained Infertility (UEI), High-Density Lipoprotein (HDL), Paraoxonase (PON), Myeloperoxidase (MPO), In Vitro Fertilization (IVF), Intra Cytoplasmic Sperm Injection (ICSI), Body Mass Index (BMI), Severe Ovarian Hyperstimulation Syndrome (OHSS), Day 3 (D3), Metaphase II (MII), Blastocyst Quality Scoring (BQS), Controlled Ovarian Stimulation (COS), Gonadotropin-Releasing Hormone (GnRH), Oocyte Pick Up (OPU), Coefficient of Variation (CV), Standard Deviation (SD), Follicle-Stimulating Hormone (FSH), Luteinizing Hormone (LH) and Estradiol (E2), Embryo Transfer (ET), Reactive Oxygen Species (ROS).
